# SIRT1 contributes to neuroendocrine differentiation of prostate cancer

**DOI:** 10.18632/oncotarget.23111

**Published:** 2017-12-11

**Authors:** Lin Ruan, Lei Wang, Xiaosong Wang, Ming He, Xiaoguang Yao

**Affiliations:** ^1^ Hebei Key Laboratory of Integrative Medicine on Liver-Kidney Patterns, Hebei University of Chinese Medicine, Shijiazhuang, China; ^2^ Department of Nephrology, The First Hospital of Hebei Medical University, Shijiazhuang, China; ^3^ Department of Urology, The First Hospital of Hebei Medical University, Shijiazhuang, China; ^4^ College of Integrative Medicine, Hebei University of Chinese Medicine, Shijiazhuang, China

**Keywords:** prostate cancer, SIRT1, neuroendocrine, N-Myc, Akt

## Abstract

The epigenetic factor SIRT1 can promote prostate cancer progression, but it is unclear whether SIRT1 contributes to neuroendocrine differentiation. In this study, we showed that androgen deprivation can induce reactive oxygen species production and that reactive oxygen species, in turn, activate SIRT1 expression. The increased SIRT1 expression induces neuroendocrine differentiation of prostate cancer cells by activating the Akt pathway. In addition, the interaction between Akt and SIRT1 is independent of N-Myc and can drive the development of neuroendocrine prostate cancer when N-Myc is blocked. Furthermore, SIRT1 facilitates tumor maintenance, and targeting SIRT1 may reduce the tumor burden during androgen deprivation. Our findings suggest that SIRT1 is a potential target for therapeutic intervention.

## INTRODUCTION

Prostate cancer (Pca) is the most common cancer among men and the second leading cause of death from cancer in the US [1]. Most patients with clinically localized Pca receive curative treatments, including radical prostatectomy or radiotherapy. Subsequently, between 20% and 40% of these patients eventually experience biochemical recurrence [2]. Once recurrence is diagnosed, these patients receive androgen deprivation therapy (ADT). Although ADT can prolong patients’ overall survival and decrease their tumor burden, all patients eventually become resistant to ADT, developing a condition called castration-resistant Pca (CRPC) [3]. A previous study reported that CRPC, as shown by disease progression, will develop after 12-18 months of ADT [4].

Therefore, understanding the molecular mechanisms leading to CRPC and identifying related molecular pathways are important in developing more effective treatments for CRPC. In recent years, many studies have found that ADT promotes the neuroendocrine differentiation (NED) of Pca cells, and cells that have undergone NED are not sensitive to hormone therapy or chemotherapy [5–6]. Therefore, NED should be considered a critical event in the development of CRPC from androgen-dependent Pca cells. If NED can be targeted and blocked, Pca cells may be eliminated by ADT combined with other appropriate therapies. In recent years, several studies have reported that the Akt pathway drives the NED of Pca [7], and the Akt pathway can be activated by multiple stimuli, including cytokines, androgen receptors, oncogenes, and epigenetic factors; therefore, Akt plays a key role in the NED of Pca. Lee JK et al. [8] suggested that N-Myc can increase Akt expression and thereby contribute to NED. *in vitro*, some studies have found that both interleukin-6 (IL-6) [9] and epidermal growth factor (EGF) [10] can activate Akt and its pathway to promote Pca progression. However, the interaction between epigenetic factors and Akt is still not well understood, and it remains unknown whether epigenetic factors can activate Akt expression independently in NED of Pca.

SIRT1, an epigenetic factor that may be associated with Pca, belongs to a family of histone deacetylases that are ubiquitously expressed in different tissues, localized in the nucleus and cytoplasm. SIRT1 is involved in cell survival, apoptosis, autophagy, and metabolism. However, the role of SIRT1 in cancer remains controversial; SIRT1 has been shown to play both a pro- and an anti-tumor role in cancer bio-behavior [11–12]. Some research has found that SIRT1 can inhibit NF-κB activation and facilitate cancer cell apoptosis [13], but other studies reported that SIRT1 contributes to cell autophagy and then promotes cell survival [14–15]. SIRT1 was also found to be active in Pca tumor progression. Lim JH et al. reported that SIRT1 can deacetylate HIF-1α and thereby promote cancer progression [16]; Cui Y et al. noted that knockdown of the SIRT1 gene in PC-3 cells suppressed the movement, migration, and invasion of the cells [17]. However, it is unclear whether SIRT1 is involved in NED of Pca cells. In our study, we performed a bioinformatics analysis of the GEO (Gene Expression Omnibus) dataset and found that multiple epigenetic factors were increased in neuroendocrine Pcas, including SIRT1. *in vitro* experiments showed that SIRT1 was increased in Pca cells with ADT and that enhanced SIRT1 expression increased the levels of NED biomarkers. We also found that SIRT1 can activate Akt directly. In addition, further investigations revealed that upregulation of SIRT1 may be induced by reactive oxygen species (ROS) production following ADT.

## RESULTS

### Elevated SIRT1 expression in Pca and neuroendocrine Pca

We searched the GEO database, identified data regarding neuroendocrine Pca resistance to androgen deprivation, and included these data—GSE66851—in our study. This dataset involved 3 samples of LNCaP cells cultured under ADT until NED development. At that point, RNA was extracted from all the samples for gene expression profiles. Reanalysis of these data on a GPL6244 platform showed that multiple genes encoding epigenetic factors, including MID1, SIRT1, MAP1B, APLP1, and AMIGO2, were upregulated under the ADT condition (Supplementary Table 1). Specifically, the post-treatment level of SIRT1 expression was 1.67-fold higher than the pretreatment level (p<0.01).

**Figure 1:**
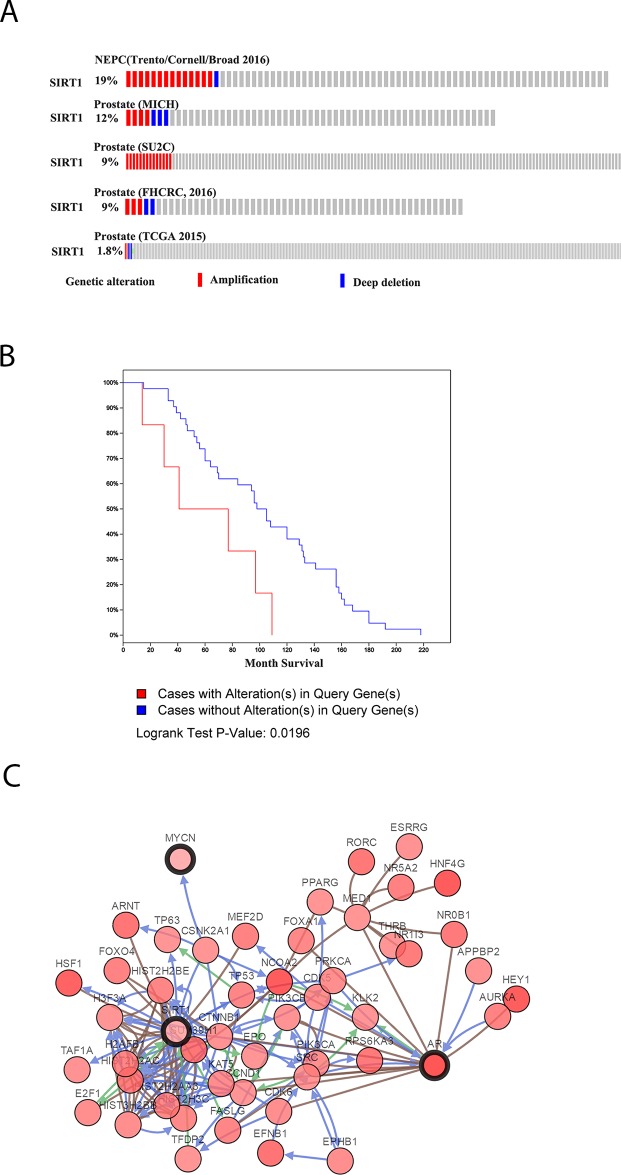
SIRT1 expression levels in Pca as recorded in TCGA database **(A)** SIRT1 expression in some published Pca datasets, including Neuroendocrine Prostate Cancer (Trento/Cornell/Broad 2016) [18], Prostate (MICH) [20], Prostate (SU2C) [19], Prostate (FHCRC, 2016) [54], and Prostate (TCGA 2015) [55]. **(B)** Survival analysis of Pca patients with SIRT1 in the dataset Prostate Adenocarcinoma, Metastatic (Michigan, Nature 2012) [20]. **(C)** Pathway analysis of SIRT1 in Pca based on the dataset Neuroendocrine Prostate Cancer (Trento/Cornell/Broad 2016) [18].

In The Cancer Genome Atlas (TCGA) database, SIRT1 amplification was widely found in 8 of 12 datasets. In the dataset of neuroendocrine Pca published by Trento in 2016 [18], SIRT1 amplification was found in 19% of 77 cases. Furthermore, in a metastatic Pca dataset published by Robinson et al. in 2015 [19], SIRT1 amplification was identified in 9% of 150 cases (Figure 1A). Moreover, in another metastatic Pca dataset, the cases with SIRT1 amplification had significantly shorter survival (p<0.05) [20] (Figure 1B). Pathway and network analyses of SIRT1 were also generated based on neuroendocrine Pca cancer from TCGA database (Figure 1C), and we found that SIRT1 could promote Pca progression via multiple pathways, including the HIF-1, SMAD4, and Akt pathways. The Akt pathway can promote NED and cancer progression. Therefore, we created a model of neuroendocrine Pca cells *in vitro* by culturing LNCaP cells under androgen deprivation conditions, and then, we investigated the effect of SIRT1 on NED development.

### ADT induces SIRT1 overexpression and NED development

**Figure 2:**
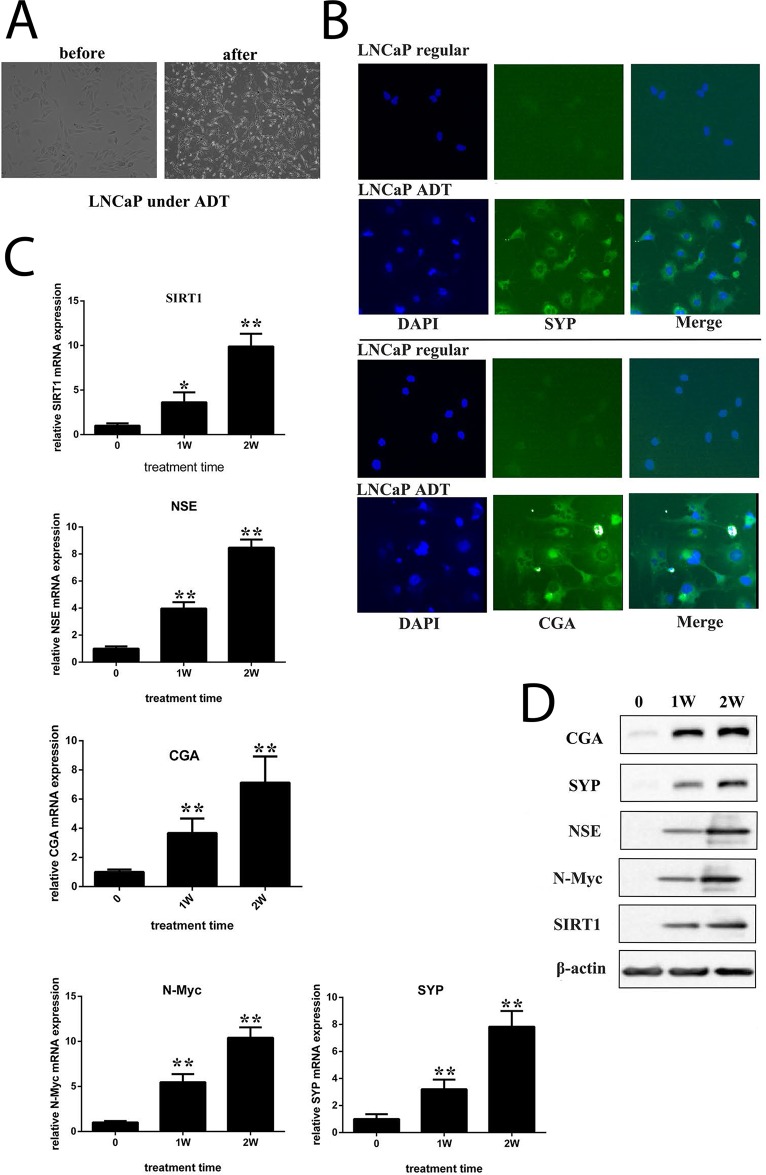
ADT induced NED of LNCaP cells *in vitro* **(A)** Images showing neuroendocrine changes in LNCaP cells cultured under ADT conditions for 2 weeks (100x magnification). **(B)** Fluorescent images of LNCaP cells that were maintained in either the regular medium or ADT medium for approximately 4 weeks (200x magnification). Cells were incubated with antibody against SYP or CGA. The expression of SYP or CGA was visualized under a fluorescence microscope. **(C)** and **(D)** mRNA levels and protein levels of candidate genes were measured in cells cultured under different durations of ADT. Three independent experiments were performed by real-time qPCR, and the data are presented as the mean±SD (columns, mean of three different experiments; bars, SD). ^*^: p<0.05; ^**^: p<0.01, by Student’s *t*-test.

LNCaP cells are an androgen-sensitive cell line carrying a mutation in the gene encoding AR. We cultured LNCaP cells *in vitro* under androgen-deprived conditions. After approximately 4 weeks of culture, the cells transformed morphologically into neuroendocrine-like cells (Figure 2A), with increased neurite length, multipolar shape, and small cell bodies. Immunofluorescence indicated the expression of synaptophysin (SYP) or chromogranin (CGA), both of which are neuroendocrine biomarkers (Figure 2B and Supplementary Figure 1A). Immunoblots showed that the expression of N-Myc and SIRT1 increased during the course of androgen deprivation, accompanied by neuron-specific enolase (NSE), SYP and CGA expression (Figure 2C and 2D). Additionally, SIRT1 activity increased in LNCaP cells under ADT conditions (Supplementary Figure 1B). All these data indicate that SIRT1 may contribute to NED. Moreover, we cultured LAPC4 cells under ADT conditions. LAPC4 cells are an androgen-dependent cell line carrying a WT gene encoding AR. The results indicated that after 1 week of culture in ADT conditions, the expression levels of all 3 neuroendocrine biomarkers (NSE, SYP and CGA) were increased, as was the SIRT1 expression level (Supplementary Figure 2A-2E).

**Figure 3:**
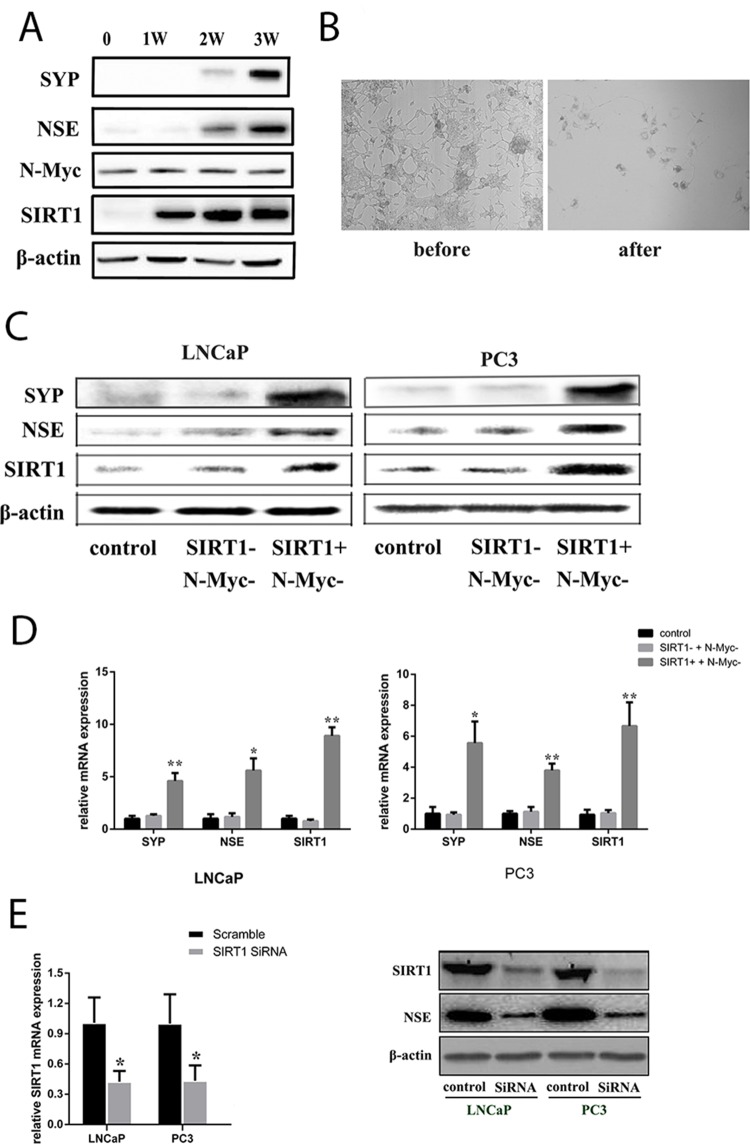
SIRT1 upregulation contributed to NED of Pca cells under ADT conditions **(A)** Expression of candidate proteins in LNCaP cells under ADT combined with an N-Myc/Aurora complex inhibitor (CD532) treatment *in vitro*. The NSE level increased over the time course of ADT, as did the SIRT1 level, but the N-Myc level remained almost constant due to CD532. **(B)** Images showing neuroendocrine changes in LNCaP cells under ADT+CD532 (100x magnification). **(C)** and **(D)** mRNA levels and protein expression levels of the candidate genes (SIRT1, SYP, NSE) in Pca cells (LNCaP and PC3) after treatment with CD532 combined with a SIRT1 activator (SRT1720) or inhibitor (ex527) for 3 days. **(E)** Knockdown of SIRT1 expression using siRNA decreased NSE expression. RNA was extracted from Pca cells transfected with SIRT1 siRNA or scrambled control siRNA for 3 days and analyzed via real-time qPCR. SIRT1 and NSE protein expression level decreased significantly after SIRT1 expression was knocked down in Pca cells (LNCaP, PC3). Three independent experiments were performed by real-time qPCR, and the data are presented as the mean±SD (columns, mean of three different experiments; bars, SD). ^*^: p<0.05; ^**^: p<0.01, by Student’s *t*-test.

Then, to investigate whether NED can occur in LNCaP without N-Myc, we subjected LNCaP cells to androgen deprivation conditions combined with an N-Myc/Aurora complex inhibitor (CD532). Cell morphology was observed, and the results indicated that NED biomarkers still increased with treatment. Blockage of the N-Myc pathway could delay the increase in NED biomarkers for a short period (Figure 3A). However, LNCaP cells eventually transformed into neuroendocrine cells, and the NSE protein level increased, accompanied by increased levels of SIRT1 (Figure 3B). Therefore, these data indicated that SIRT1 could promote NED independently, instead of through interaction with the N-Myc protein. To verify whether SIRT1 expression could induce NED, we treated LNCaP cells and PC3 cells with a SIRT1 activator combined with an N-Myc inhibitor, and the results showed that NED biomarker expression was increased (Figure 3C and 3D, Supplementary Figure 3A and 3B). In addition, almost no changes in NED biomarker expression were found in cells treated with the SIRT1 inhibitor and the N-Myc inhibitor. However, when SIRT1 was knocked down by siRNA, the expression level of NSE was significantly decreased in Pca cells (LNCaP, PC3; see Figure 3E). Besides, NCI-H660 is a neuroendocrine prostate cancer cell line with SIRT1 neuroendocrine biomarkers expression. But NSE expression decreased correspondingly when SIRT1 was knocked down by SiRNA (Supplementary Figure 4).

### SIRT1 promotes NED via SIRT1-Akt signaling

**Figure 4:**
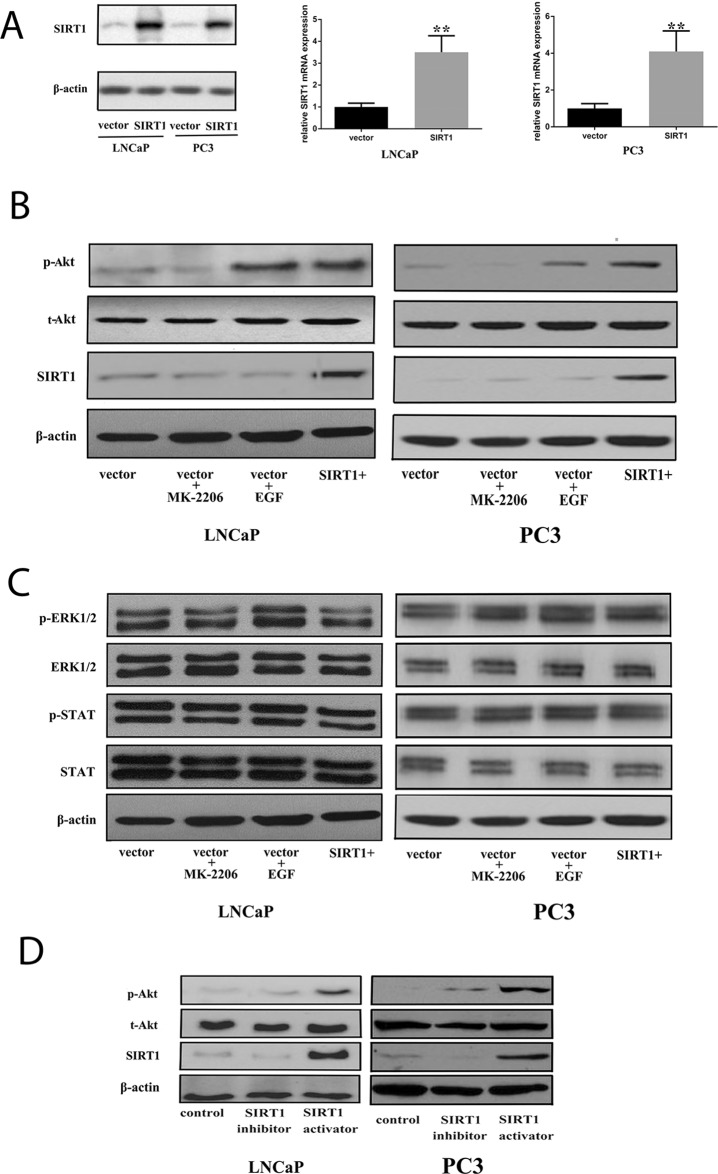
Increased expression of SIRT1 can activate the Akt pathway **(A)** Pca cells (LNCaP and PC3) were transfected with an empty vector or one encoding SIRT1. After 36 h of transfection, cell pellets were lysed and prepared for real-time qPCR analysis or western blot analysis using specific antibodies. Three independent experiments were performed by real-time qPCR, and the data are presented as the mean±SD (columns, mean of three different experiments; bars, SD). ^*^: p<0.05; ^**^: p<0.01, by Student’s *t*-test. **(B)** Akt was phosphorylated and activated in Pca cells after transfection with SIRT1 for 3 days. Expression of phospho-Akt increased in cells with SIRT1 overexpression as well as cells treated with the positive control EGF (an Akt activator, 20 ng/ml), while increased phospho-Akt was not found in cells transfected with the vector or in cells transfected with the vector and treated with the negative control MK2206 (a phospho-Akt inhibitor, 10 μM). **(C)** There was no statistically significant change in ERK1/2 or STAT activation in cells with SIRT1 overexpression. **(D)** Akt was phosphorylated and activated in Pca cells after 3 days of treatment with a SIRT1 activator. p-Akt: phospho-Akt; t-Akt: total Akt; vector: transfection with vector only; vector + MK2206, cells transfected with vector and treated with MK2206; vector + EGF, cells transfected with vector and treated with EGF; SIRT1+: transfection with SIRT1; SIRT1 activator: SRT1720; SIRT1 inhibitor: ex527.

We generated Pca cells with enforced expression of SIRT1 by transfection (Figure 4A). Subsequently, we used western blots to investigate protein expression in several pathways under this condition. The results showed that SIRT1 transfection increased the phospho-Akt (p-Akt) level but did not affect other pathways, such as ERK and STAT (Figure 4B and 4C), in both androgen-sensitive Pca cells (LNCaP) and androgen-independent Pca cells (PC3). In addition, a SIRT1 activator significantly increased p-Akt expression, while a SIRT1 inhibitor decreased the p-Akt expression level compared with that of the vehicle treatment group (Figure 4D and Supplementary Figure 2F).

**Figure 5:**
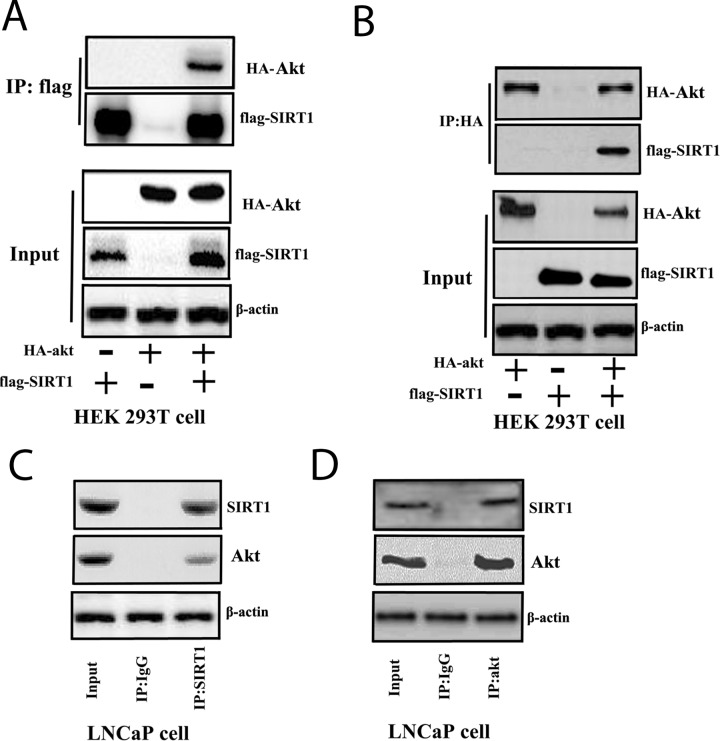
SIRT1 interacted with Akt in cells **(A)** and **(B)** FLAG-SIRT1: interaction with HA-Akt in HEK 293T cells. Flag-SIRT1 and HA-Akt were transfected individually or co-transfected into HEK 293T cells for 3 days. The cell lysates were immunoprecipitated with anti-FLAG (Figure 5A) or anti-HA (Figure 5B) antibodies. Then, the immunoprecipitates were subjected to western blot analysis. **(C)** and **(D)** SIRT1 interacted with Akt in LNCaP cells. LNCaP cells were cultured and lysed for IP with anti-SIRT1 (Figure 5C) or anti-Akt (Figure 5D) antibodies or a control IgG antibody. Then, western blot analysis was performed.

Furthermore, we investigated the interaction between SIRT1 and Akt. We overexpressed FLAG-tagged SIRT1 and HA-tagged Akt in HEK293A cells and performed an immunoprecipitation (IP) assay with anti-FLAG or anti-HA antibodies to determine the interaction between SIRT1 and Akt. The results identified a physical interaction between SIRT1 and Akt (Figure 5A and 5B). SIRT1 deacetylated Akt and promoted phosphorylation of Akt (Supplementary Figure 5A). Furthermore, enhanced SIRT1 expression in cells can activate Akt pathway activity through FOXO reporter system (Supplementary Figure 5B). We further examined the interaction between endogenous SIRT1 and Akt in Pca cells. We found that endogenous SIRT1 and Akt interacted with each other in LNCaP cells (Figure 5C and 5D).

### Production of ROS promotes SIRT1 expression under androgen deprivation conditions

**Figure 6:**
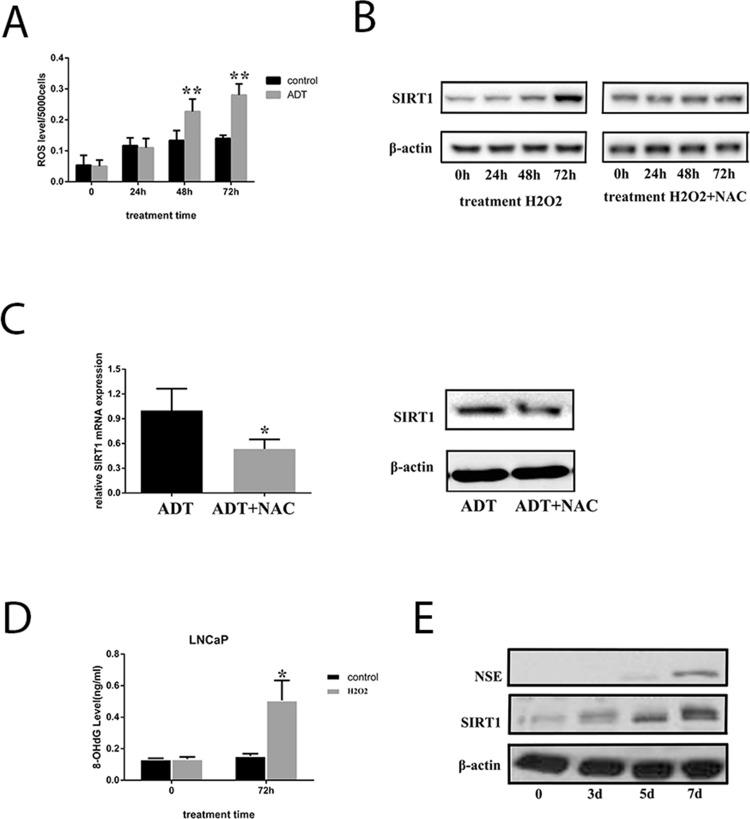
ADT increased SIRT1 expression by inducing ROS levels in LNCaP cells **(A)** ROS levels in LNCaP cells increased in a time-dependent manner under ADT. In each group (control or ADT), all the ROS levels were compared with the value before treatment (time point 0). **(B)** SIRT1 expression in the cells increased in a time-dependent manner with H2O2 treatment, and the upregulation of SIRT1 expression can be blocked by NAC. **(C)** Both mRNA (left) and protein (right) expression of SIRT1 was inhibited in LNCaP cells under ADT combined with NAC. Cells were cultured under ADT alone or in combination with NAC for 3 days, after which cells were collected. Three independent experiments were performed by real-time qPCR, and data are presented as the mean±SD (columns, mean of three different experiments; bars, SD). ^*^: p<0.05; ^**^: p<0.01, by Student’s *t*-test. **(D)** The 8-OHdG levels were measured after LNCaP cells were cultured under ADT for 72 h. Columns, mean of three different experiments; bars, SD. ADT treatment significantly increased 8-OHdG levels (^*^, p < 0.05 by Student’s *t*-test). **(E)** ROS promoted both SIRT1 and NSE expression in LNCaP cells. The cells were treated with H2O2 for the indicated period, and then, the cell pellets were lysed and prepared for western blot analysis with specific antibodies. The results indicated that NSE levels increased after LNCaP cells were treated with H2O2 for 7 days.

We cultured LNCaP cells under androgen deprivation conditions for 3 days, and production of ROS was measured. The data indicated that ROS increased significantly in LNCaP cells (Figure 6A and Supplementary Figure 6); thus, we hypothesized that androgen deprivation therapy could induce ROS production. Increased SIRT1 was also observed in the cells when they were cultured under androgen deprivation; therefore, ROS production is a candidate explanation for the increased SIRT1 (Figure 6B). When LNCaP cells were treated with H_2_O_2_ (a type of ROS), SIRT1 expression was increased, but SIRT1 failed to increase when treated with an anti-ROS reagent (N-acetylcysteine, NAC) combined with H_2_O_2_. Similarly, the SIRT1 level was much lower in LNCaP cells treated with NAC under ADT than in LNCaP cells under ADT only (Figure 6C). Furthermore, DNA injury by ROS could activate SIRT1 expression. In our study, the results showed that H_2_O_2_ treatment increased the 8-OHdG levels, a biomarker of DNA injury, in LNCaP cells and LAPC4 cells (Figure 6D and Supplementary Figure 7). In addition, H_2_O_2_ treatment increased expression of both SIRT1 and NSE in LNCaP cells (Figure 6E). Multiple studies have found that DNA injury in the cells activates SIRT1 expression to facilitate cell survival. Therefore, we speculated that androgen deprivation could induce production of ROS, and ROS could increase SIRT1 expression via DNA injury.

### Xenograft study

**Figure 7:**
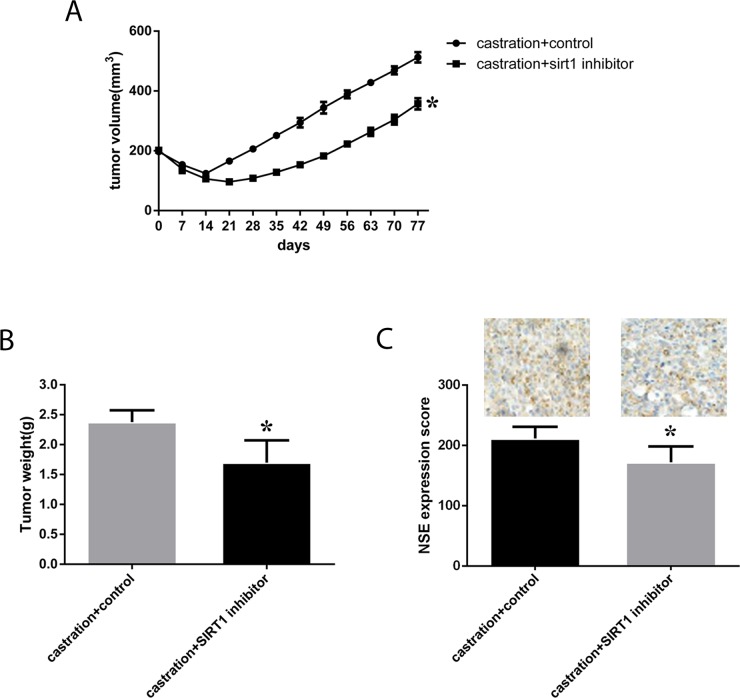
Effect of a SIRT1 inhibitor (ex527) on the progression and growth of xenografts in mice LNCaP cells were cultured under ADT conditions until neuroendocrine transformation developed. Then, male SCID mice were injected subcutaneously with cells in Matrigel. After 4-6 weeks, mice with tumors (200 mm3) were surgically castrated. The castrated mice were injected IP with ex527 at 10 mg/mouse/week for approximately 90 days. The control group was injected with the vehicle. Tumor volume and tumor weight were monitored and are presented here as the mean±SD. **(A)** and **(B)** Growth curves and tumor weight of xenografts in each group were calculated, and statistical analysis was performed. **(C)** Quantitative IHC analysis and representative microscopic fields of NSE (200x magnification). In the columns, data are expressed as the mean±SD, generated as described in the Materials and methods. ^*^: p<0.05 by two-tailed Student’s *t*-test.

We also investigated the effect of SIRT1 expression *in vivo*. Eight-week-old male nude mice were subcutaneously injected with neuroendocrine-transformed LNCaP cells, and then, tumor volumes were monitored every 3 days. Surgical castration was performed when the tumor volume reached 200 mm^3^, followed by treatment with the SIRT1 inhibitor ex527, and further growth of the tumors was monitored. Castration resulted in a significant regression of the tumors, from more than 200 mm^3^ to 100 mm^3^ two weeks after castration, and then, the tumors started to regrow. Overall, we found that in the group of mice subjected to castration and ex527, tumor growth was much slower than in the control groups (p=0.014, see in Figure 7), and tumor weight was much lower in mice with SIRT1 inhibitor treatment. Moreover, we investigated the expression of NSE in tumors, and the results showed that the NSE expression level was much lower in mice treated with the SIRT1 inhibitor.

## DISCUSSION

Neuroendocrine cells are one of three types of epithelial cells in normal prostate tissue, where they constitute <1% of total epithelial cells and have an unclear physiological role [21]. In prostate adenocarcinoma, an increased number of neuroendocrine cells is observed, and this change is always associated with poor prognosis, including frequent metastasis, relatively low serum prostate-specific antigen (PSA) level, and resistance to androgen ablation [22]. Therefore, identifying novel elements involved in NED is critical for understanding the mechanism of Pca progression and developing new drug targets for the treatment. The prevailing hypothesis is that Pca undergoes NED, especially under the selective pressure of androgen deprivation [23–24]. Our results functionally demonstrated that SIRT1 can arise from ROS production in Pca cells responding to ADT. Further, SIRT1 upregulation can activate Akt expression, which, in turn, promotes NED.

Taking advantage of published gene expression from GEO, we downloaded and reanalyzed raw data from both LNCaP cells and LNCaP-NED cells. Focusing on epigenetic factors involved in NED, we discovered that multiple epigenetic factors, including SIRT1, are clustered and that their expression is upregulated during NED. Importantly, amplification of SIRT1 was evident in neuroendocrine prostate tumors from TCGA database, providing validation of our expression analysis.

SIRT1 is an NAD^+^-dependent histone deacetylase that regulates apoptosis in response to oxidative and genotoxic stress [25–26]. From a mechanistic standpoint, our study revealed that ADT can increase ROS production in androgen-sensitive LNCaP cells and that ROS can then activate SIRT1 expression. SIRT1 activates the Akt pathway via a direct physical interaction, instead of acting through N-Myc. The role of N-Myc in Pca has been recognized and reported by multiple studies, and its amplification is often associated with Pca progression, poor clinical outcome [8, 27–29], and CRPC development [30]. Notably, N-Myc was shown to induce NED by activating the Akt pathway in Pca cells [8]. However, our study focused on whether NED would develop after N-Myc was blocked. The results reveal that there is an alternative way to activate Akt via the epigenetic factor SIRT1. In addition, we investigated whether SIRT1 activated other pathways in Pca when treated with ADT. Multiple studies have reported that both the ERK pathway and STAT pathway are involved in Pca progression. However, in our study, the results showed that neither ERK nor STAT was activated by SIRT1.

Recent studies have indicated that SIRT1 is expressed in various types of cancers, including colon cancer [31], breast cancer [32], Pca [33], squamous cell carcinoma [34], and lung cancer [35]. Additionally, increased expression of SIRT1 has been reported to promote Pca progression. Debelec-Butuner B et al. found that SIRT1 expression was activated in conditioned medium without androgens [36], and activated SIRT1 was involved in the regulation of multiple pathways in Pca cells. Zhou ZW et al. found that plumbagin promoted Pca cell apoptosis by blocking the Akt pathway, and this inhibitory effect on Akt pathway occurred via downregulation of SIRT1 expression [37]. Acetylation can prevent localization and phosphorylation of Akt by blocking the binding of Akt to PIP3. However, as a deacetylase, SIRT1 can deacetylate Akt and enhance its binding to PIP3, promoting their activation [38]. In this study, a SIRT1-Akt interaction was demonstrated, as Akt was efficiently pulled down with FLAG-SIRT1. More importantly, increased SIRT1 expression in cells enhanced Akt phosphorylation, and Akt expression was correlated with NED biomarker levels. Activation of the Akt pathway promotes cancer cell proliferation, epithelial-mesenchymal transition, NED, and progression. Therefore, we suggest that SIRT1 could be an alternative contributor to NED through activation of the Akt pathway.

In Pca cells, ADT can induce ROS [39–40], and subsequently, ROS-related stress can induce genome instability and gene expression changes to adapt to changing circumstances. Other research has shown that castration induces oxidative stress in the rat prostate by significantly upregulating ROS-generating NADPH oxidases and downregulating ROS-detoxifying enzymes [41]. Additionally, in human Pca biopsy tissues, ADT can decrease the mRNA level of manganese superoxide dismutase (MnSOD), which is one of the major ROS scavengers [42]. ROS generated from either endogenous or external sources contribute to a wide range of biological mechanisms. Our data showed that the ROS level significantly increased in LNCaP cells under ADT. Moreover, *in vitro*, H_2_O_2_ (ROS product) increased the SIRT1 level in Pca cells, but SIRT1 was blocked when cells were treated with H_2_O_2_ combined with the anti-ROS reagent NAC. Therefore, SIRT1, as an epigenetic factor, may be activated by ROS production under ADT to facilitate cancer cell survival through activation of Akt. Similarly, Nasrin N *et al.* suggested that oxidative stress triggers the interaction of JNK1 with SIRT1 and that JNK1 consequently phosphorylates SIRT1 and then increases SIRT1 activity [43]. Additionally, oxidant-induced SIRT1 overexpression inhibits p53-mediated nuclear transactivation and blocks cell apoptosis [44].

In sum, our study showed that ADT leads to production of ROS, which in turn induce NED through activated SIRT1-Akt. We discovered a novel regulatory axis that involves ROS, SIRT1, Akt and their functions during NED. The high expression and NED function of SIRT1 in Pca cells support its candidacy as a therapeutic target for patients with Pca.

## MATERIALS AND METHODS

### Online database analysis

Online data regarding Pca were obtained from GEO (http://www.ncbi.nlm.nih.gov/geo/) and TCGA to identify datasets suitable for reanalysis. In the GEO database, the following keywords were used: ‘prostate cancer’, ‘androgen deprivation’, and ‘neuroendocrine’. Five datasets were identified. After analysis of the abstracts of the datasets, only the dataset GSE66851 (GPL6244, Affymetrix Human Gene 1.0 ST Array) was selected for use. Next, we downloaded the datasets (CEL files) and prepared the server for data analysis with R statistical software (www.r-project.org), using the Bioconductor library. Analysis of this dataset, which included information regarding genes related to epigenetic factors, was subsequently performed. Gene expression levels were quantified after analysis.

In TCGA database, using the online analysis platform cBioPortal [45–46], we analyzed the mutation and CNA of SIRT1 in 12 Pca datasets, and a gene expression network was generated. Additionally, the relationship between SIRT1 expression and survival was analyzed.

### Cell culture

Pca cell lines (LNCaP, LAPC-4, NCI-H660 and PC3) and HEK 293T cells were purchased from the American Type Culture Collection (ATCC) and were maintained in RPMI 1640 (Invitrogen), HITES (Sigma) or DMEM (HyClone), which were supplemented with 10% ultracentrifuged fetal bovine serum (FBS; Invitrogen), 1 mM sodium pyruvate (Invitrogen), penicillin (100 U/ml; Invitrogen) and streptomycin (10 mg/ml; Invitrogen) at 37 °C in a humified atmosphere with 5% CO_2_. In addition, 0.05% trypsin and 0.02% ethylenediamine tetra-acetic acid (HyClone) were also used in the cell culture. When LNCaP and LAPC-4 cells were cultured under ADT conditions, the androgen-deprived medium was routinely prepared by adding 10% charcoal-stripped serum instead of untreated FBS.

### Compound preparation and *in vitro* treatment

A SIRT1 activator [47–48] (SRT1720, 4 μM) and a SIRT1 inhibitor [49] (ex527, 0.4 μM) were purchased from Selleck Chemicals and dissolved in DMSO. An N-Myc/Aurora complex inhibitor (CD532, 0.5 μM) was purchased from Millipore and dissolved in DMSO. H_2_O_2_ and NAC were applied to Pca cells at concentrations of 1 mM and 5 mM, respectively [40]. EGF and a highly selective inhibitor of Akt1/2/3 (mk2206) were purchased from Selleck Chemicals and applied to the cells at 20 ng/ml [50] and 10 μM [51], respectively.

### Plasmid construction and transfection

Full-length cDNAs of human SIRT1 (Origene) and wild type Akt (Origene) were sequenced and subcloned into a pcDNA3.1 expression vector. The expression construct pcDNA3.1 contains a Flag or HA tag. A lentiviral vector expressing tagged SIRT1 or Akt was generated using the ViraPower^TM^ T-REx^TM^ system following the instructions from the manufacturer (Invitrogen). HEK 293T cells were transfected using the calcium phosphate precipitation method, and Pca cells were transfected using Fu GENE6 Transfection Reagent (Roche Applied Sciences, US).

### siRNA transfection

Cells were transfected with anti-SIRT1 siRNA or a scrambled probe, which was used as a control (Santa Cruz, CA, USA), at a final concentration of 40 nM, using Lipofectamine 2000 (Invitrogen), in accordance with the manufacturer’s instructions. Transfection efficiency was validated via real-time qRT-PCR, which was used to measure SIRT1 expression.

### Immunofluorescence

LNCaP cells were seeded on coverslips and then cultured under ADT until NED development. For detection of SYP or CGA, LNCaP cells were fixed with 4% formaldehyde for 10 min and blocked for 20 min in PBS containing 1% (wt/vol) BSA–0.1% (vol/vol) Triton X-100. Then, a primary antibody (rabbit monoclonal anti-SYP (Abcam) or rabbit monoclonal anti-CGA (Abcam) was diluted 1:200 and incubated at 4 °C overnight. Subsequently, the cells were incubated at room temperature for 1 h with anti-rabbit IgG antibody (Life Technology, 4 μg/mL) at room temperature and then mounted in antifade mounting medium with DAPI. The negative controls were cells cultured with regular culture medium without ADT. Fluorescent images were acquired on a fluorescence microscope (Leica Microsystems, Mannheim, Germany).

### IP and western blotting

Cell pellets were lysed using RIPA buffer with a proteinase inhibitor cocktail, and IP analysis was performed using standard protocols based on previously described methods [52]. Protein concentration was determined by a detergent-compatible BCA protein assay (Bio-Rad, Hercules, US). The samples were diluted to same quantities (20 μg) using loading buffer and were then loaded. Protein samples were separated by SDS–PAGE electrophoresis on 8-10% gels, and electrotransferred onto PVDF membranes (Millipore Corp.; Bedford, US) using a Mini Trans-Blot wet transfer system (Bio-Rad; Hercules, US). Membranes were blocked in TBS containing 0.1% TWEEN 20 and 5% non-fat milk for 1 h. The blots were then washed with TBS-TWEEN and incubated with primary antibody (1:5,000) overnight at 4 °C. Subsequently, the membrane was extensively washed and then incubated with HRP-linked anti-mouse IgG (1:3,000, Cell Signaling) or HRP-linked anti-rabbit IgG (1:3,000, Cell Signaling) antibodies conjugated to horseradish peroxidase for 2 h after being washed with TBS-TWEEN. Detection of antibody reactivity was performed using a Western Bright ECL western blotting detection kit (Bio-Rad Laboratories; Hercules, US). Equal sample loading was verified by detection of β-actin (Supplementary Figure 8). The primary antibodies included in the immunoblot were as follows: mouse monoclonal anti-SIRT1 (Santa Cruz), mouse monoclonal anti-N-Myc (Santa Cruz), mouse monoclonal anti-NSE (neuron-specific enolase, Santa Cruz), rabbit monoclonal anti-SYP (Thermo Fisher), rabbit monoclonal anti-Akt (Cell Signaling), rabbit monoclonal anti-phospho-Akt (Cell Signaling), rabbit monoclonal anti-STAT (Abcam), rabbit monoclonal anti-phospho-STAT (Abcam), rabbit monoclonal anti-ERK1/2 (Santa Cruz), rabbit monoclonal anti-phospho-ERK1/2 (Santa Cruz), rabbit polyclonal anti-acetyl lysine (Abcam), and mouse monoclonal anti-β-actin (Santa Cruz).

### SIRT1 activity assay

SIRT1 activity was measured using a SIRT1 Fluorescent Activity Assay Kit (Abcam) according to the manufacturer’s instructions using a fluorescent emission at 460 nm.

### PI3K/Akt pathway activity assay

PI3K/Akt pathway activity was measured using a FOXO Reporter kit (Qiagen) according to the manufacturer’s instructions. HEK-293T cells were co-transfected with FOXO-reporter with/without SIRT1 plasmid. After 72h of transfection, the luciferase activities were analyzed, and promoter activity values were normalized to that of the internal control, Renilla luciferase activities. Experiments were done in triplicates.

### Immunohistochemical staining (IHC)

Sections (5 μm) on glass slides were deparaffinized, rehydrated and then subjected to endogenous peroxidase blockage in 3% H_2_O_2_ and antigen retrieval in boiling 10% citrate buffer. Slides were blocked with goat serum and then incubated with monoclonal antibodies (1:200 dilution) against NSE (Santa Cruz) overnight at 4 °C. The slides were then incubated with a horseradish peroxidase-labeled dextran polymer coupled to an anti-mouse or anti-rabbit (Abcam, ab93705) antibody for 30 min at room temperature after 3 washes in PBS buffer. Finally, the slides were developed with diaminobenzidine for 4 min and counterstained with hematoxylin after 3 washes in PBS. Staining specificity was confirmed by processing sections from the same paraffin block and omitting the primary antibody as a negative control. As a positive control, we performed reactions with tissue sections specified by the manuals of the antibody providers. Cytoplasmic staining that was clearly distinguishable from the background was considered positive.

The slides were reviewed twice by 3 blinded investigators using a Nikon microscope. Target protein expression was graded semiquantitatively according to the staining score results, and the mean values were used for statistical analysis.

### RNA isolation and real-time qPCR

RNA was extracted using an RNeasy Mini Kit, and cDNA was generated using a reverse transcription kit (SuperScript III First-Strand Synthesis System; Invitrogen, Carlsbad, US). Candidate genes were measured using a SYBR Green-based reagent (SYBR GreenER qPCR SuperMix for iCycler; Invitrogen, Carlsbad, US) on a real-time PCR system (ABI 7900 Real-time PCR System) according to the manufacturer’s protocol. The primers for SIRT1 were as follows: F-5’-TAGCCTTGTCAGATAAGGAAGGA-3’; R-5’-ACAG CTTCACAGTCAACTTTGT-3’. The primers for SYP were as follows: F-5’-CAGTGGCTCTTTGCTAT TTTCGC-3’; R-5’-CTTGGCTTCGTTGTTGCAGC-3’. The primers for NSE were as follows: F-5’-AGCCTCTACGGGCATCTATGA-3’; R-5’-TTCTCAGT CCCATCCAACTCC-3’. The primers for CGA were as follows: F-5’-CAGCCAACGCTGCTTCTCA-3’; R-5’-GGTTCCTGTTATCCACTGGCA-3’. The primers for NSE were as follows: F-5’-AGCCTCTACGGGCAT CTATGA-3’; R-5’-TTCTCAGTCCCATCCAACTCC-3’. The primers for β-actin were as follows: F-5’-GTCTGCCTTGGTAGTGGATAATG-3’; R-5’-TCGAG GACGCCCTATCATGG-3’. All PCR reactions were run in triplicate; values were quantified with corresponding standard curves. Expression levels of SYP, NSE, and SIRT1 were normalized to the expression of β-actin. Relative quantification of gene expression was calculated using the 2^–ΔΔCt^ method.

### Nitroblue tetrazolium (NBT) assay

LNCaP Cells were seeded in 96-well plates. After the cells were cultured under ADT conditions or normal conditions (control), ROS production was detected by an NBT assay (Sigma, US). Cells were washed 3 times with PBS buffer to remove debris and then were incubated for 90 min in PBS containing 0.1% NBT. NBT is a yellow water-soluble chemical compound composed of two tetrazole moieties. NBT changes to a deep blue-colored precipitate when exposed to ROS. The cell precipitate was dissolved by sonication in 50% acetic acid, and optical absorbance was measured at 560 nm. Then, cells in each well were counted, and the results were normalized to 5000 cells in each treatment.

### DCFDA cellular ROS detection assay

A DCFDA cellular ROS detection assay (Abcam, MA, USA) was used to measure the activity of hydroxyl, peroxyl and other ROS within the cells. A total of 5 × 10^3^ cells per well were seeded on a 96-well plate. The cells were then stained with 25 μM DCFDA for 45 min at 37 °C. After being stained, the cells were cultured under ADT conditions. Finally, the fluorescent intensity was determined by fluorescence spectroscopy with maximum excitation and emission spectra of 495 and 529 nm, respectively.

### Animal xenograft study

LNCaP cells were cultured under ADT conditions until NED transformation with morphological change, and then, 1×10^6^ cells mixed with Matrigel (1:1) were injected subcutaneously into both flanks of male SCID mice (8 weeks old). The dimensions of each tumor were directly measured with calipers every 3 days, and the volume was calculated by the following formula: Tumor volume = 1/2(length × width^2^). The care and treatment of experimental animals were in accordance with institutional guidelines. When the tumor volume reached 200 mm^3^, castration was performed under isoflurane anesthesia with proper aseptic and antiseptic technique, and then, the tumors regressed. The castrated mice were randomized into 2 groups (8 xenografts/group): castration + SIRT1 inhibitor (ex527) and castration + vehicle. Mice received ex527 at 10 mg/mouse/week [53] or a vehicle control by intraperitoneal (IP) injection. Tumor volumes in both groups were monitored, and growth curves were generated. At the end of this study, the animals were sacrificed and the tumors were collected.

### Statistics

Data are expressed as the mean ± SD of three independent experiments. One-way ANOVA or Fisher’s exact test was used for statistical analysis. Differences were considered significant at p values less than 0.05.

### Compliance with ethical standards

All animal experiments followed the guidelines of the Institutional Animal Care and Use Committee of Hebei Medical University.

## SUPPLEMENTARY MATERIALS FIGURES AND TABLE




