# Supplementary material for: SIRT1 contributes to neuroendocrine differentiation of prostate cancer
Source: Oncotarget. 2017 Dec 11;9(2):2002–16. doi: 10.18632/oncotarget.23111 (PMC5788616; doi:10.18632/oncotarget.23111)
Supplement: Supplementary file 1 [file oncotarget-09-2002-s001.pdf]

SIRT1 contributes to neuroendocrine differentiation of prostate cancer

SUPPLEMENTARY MATERIALS

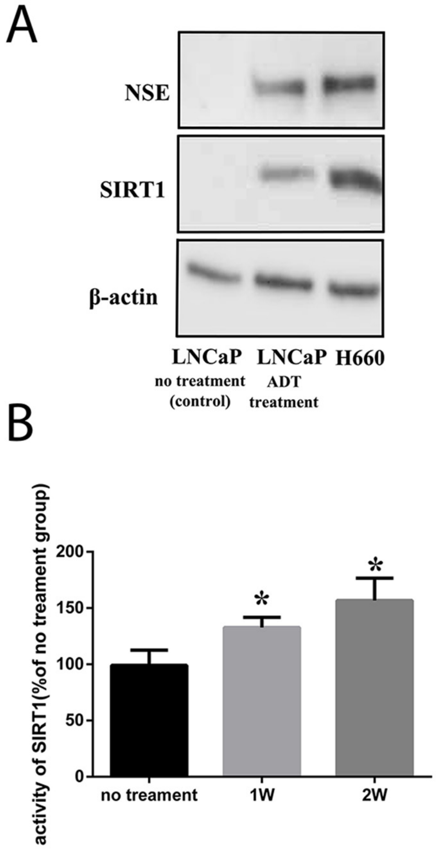

Supplementary Figure 1: SIRT1 expression and its activity in LNCaP cells under ADT conditions. (A) LNCaP cells were cultured in androgen deprivation conditions for 1 week, and their cell lysates subjected to western blot analysis. NCI-H660, a neuroendocrine prostate cancer cell line, was used as a positive control. **(B)** SIRT1 enzyme activity assay. SIRT1 activities of LNCaP cells after cultured in androgen deprivation conditions for 1 or 2 weeks.

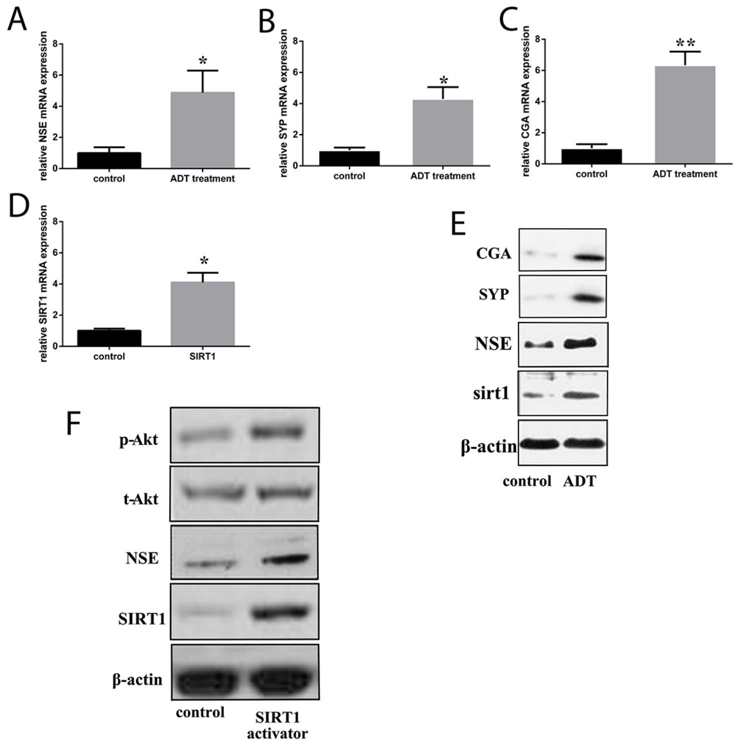

Supplementary Figure 2: ADT induced NED of LAPC4 cells *in vitro*. (A-C) mRNA levels of neuroendocrine genes (NSE, SYP and CGA) in cells cultured under ADT for 1 week. **(D)** mRNA expression level of SIRT1 in cells cultured under ADT for 1 week. Three independent experiments were performed by real-time qPCR, and the data are presented here as the mean±SD. *: $p<0.05$; **: $p<0.01$, by Student's *t*-test. **(E)** Protein expression levels of neuroendocrine genes (NSE, SYP and CGA) and SIRT1 in cells cultured under ADT for one week. **(F)** Akt was phosphorylated and activated in LAPC4 cells after 3 days of treatment with a SIRT1 activator. The SIRT1 activator also promoted SIRT1 and NSE expression.

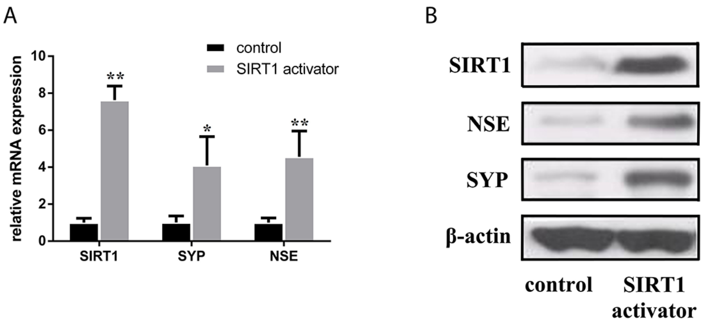

Supplementary Figure 3: mRNA **(A)** and protein **(B)** expression levels of the candidate genes (SIRT1, SYP, NSE) in LNCaP cells after 3 days of treatment with a SIRT1 activator (SRT1720). Three independent experiments were performed by real-time qPCR, and the data are presented as the mean±SD. *: $p<0.05$; **: $p<0.01$, by Student's *t*-test.

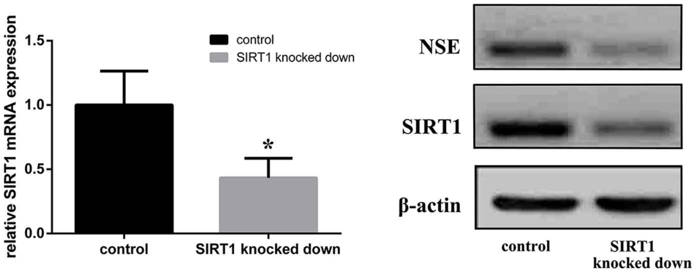

Supplementary Figure 4: Knockdown of SIRT1 expression using siRNA decreased NSE expression in NCI-H660 cells. RNA was extracted from NCI-H660 cells transfected with SIRT1 siRNA or scrambled control siRNA for 3 days and analyzed via real-time qPCR. SIRT1 and NSE protein expression level decreased significantly after SIRT1 expression was knocked down in cells. Three independent experiments were performed by real-time qPCR, and the data are presented as the mean±SD (columns, mean of three different experiments; bars, SD). *: p<0.05, by Student's *t*-test.

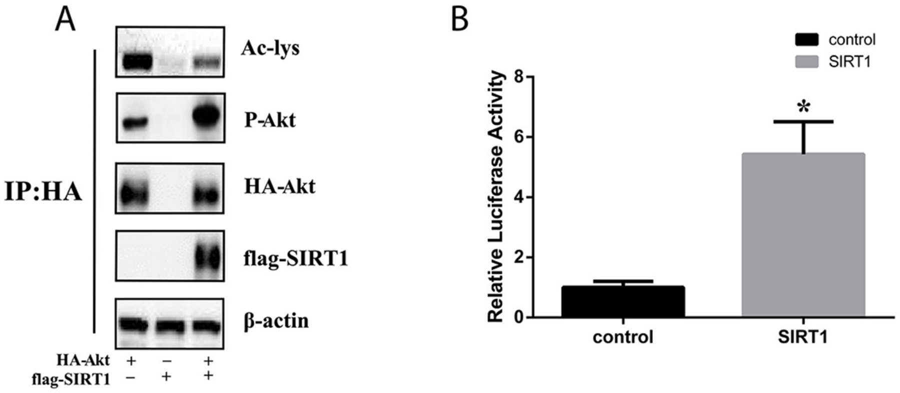

Supplementary Figure 5: SIRT1 activate Akt and its pathway. **(A)** The phosphorylation status and acetylation status of Akt immunoprecipitated from HEK293T cells were determined by immunoblotting. AC-lys: anti-acetyl lysine antibody. **(B)** Luciferase assay of FOXO reporter after co-transfection with SIRT1 expression plasmid in HEK-293T cells. Control group, FOXO reporter after co-transfection with vector, SIRT1 group, FOXO reporter after co-transfection with SIRT1 plasmid. The data are presented as the mean±SD. *: p<0.05; by Student's *t*-test.

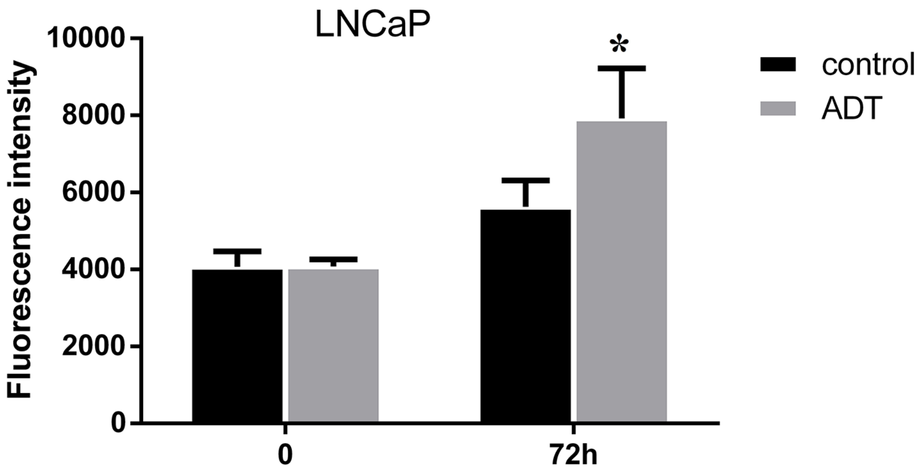

Supplementary Figure 6: ROS levels in LNCaP cells increased after 72 h under ADT conditions by DCFDA cellular ROS detection assays. In each group (control and ADT), all the ROS levels were compared with the value before treatment (time point 0) correspondingly. The data represent the mean±SD. *p<0.05 by Student's *t*-test.

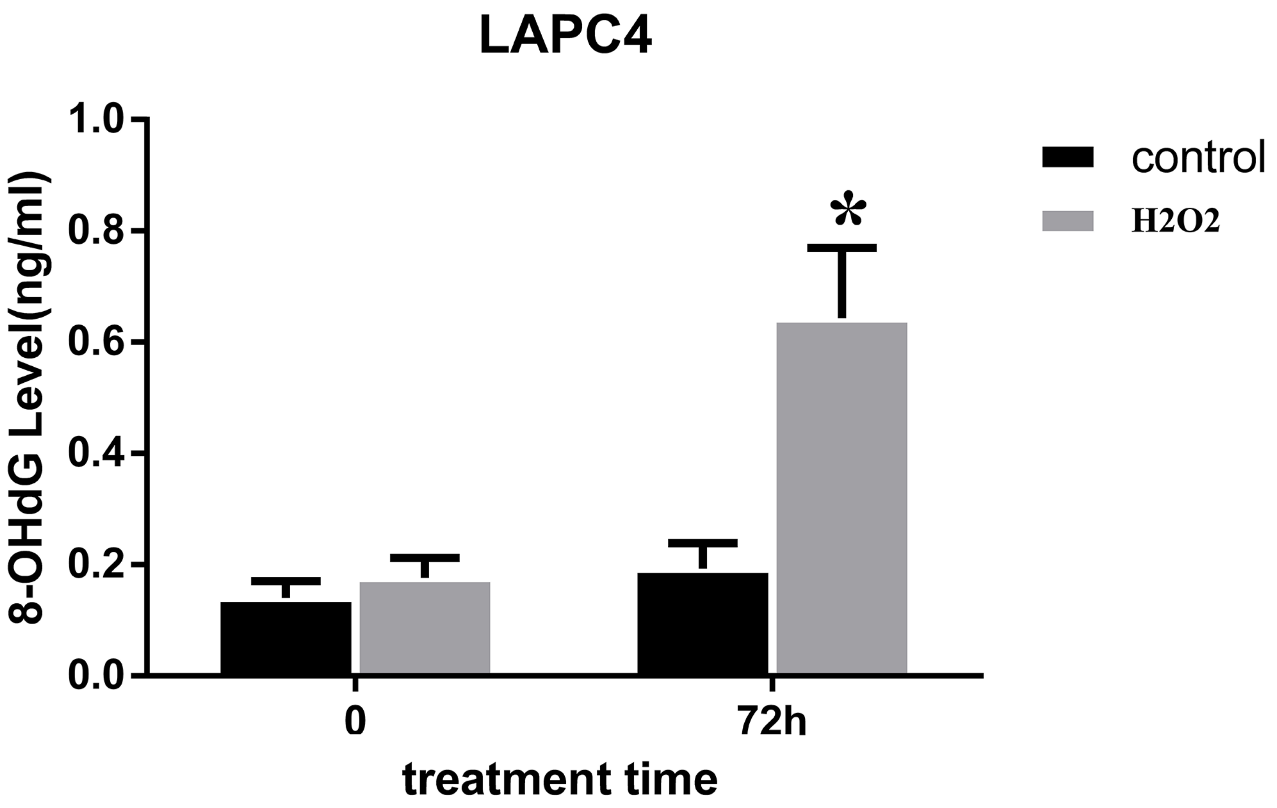

Supplementary Figure 7: The 8-OHdG levels were measured after LAPC4 cells were cultured under ADT for 72 h. 8-OHdG levels were compared with the values before treatment (time point 0) correspondingly. Columns, mean of three different experiments; bars, SD. ADT treatment significantly increased 8-OHdG levels (*, $P < 0.05$ by Student's *t*-test).

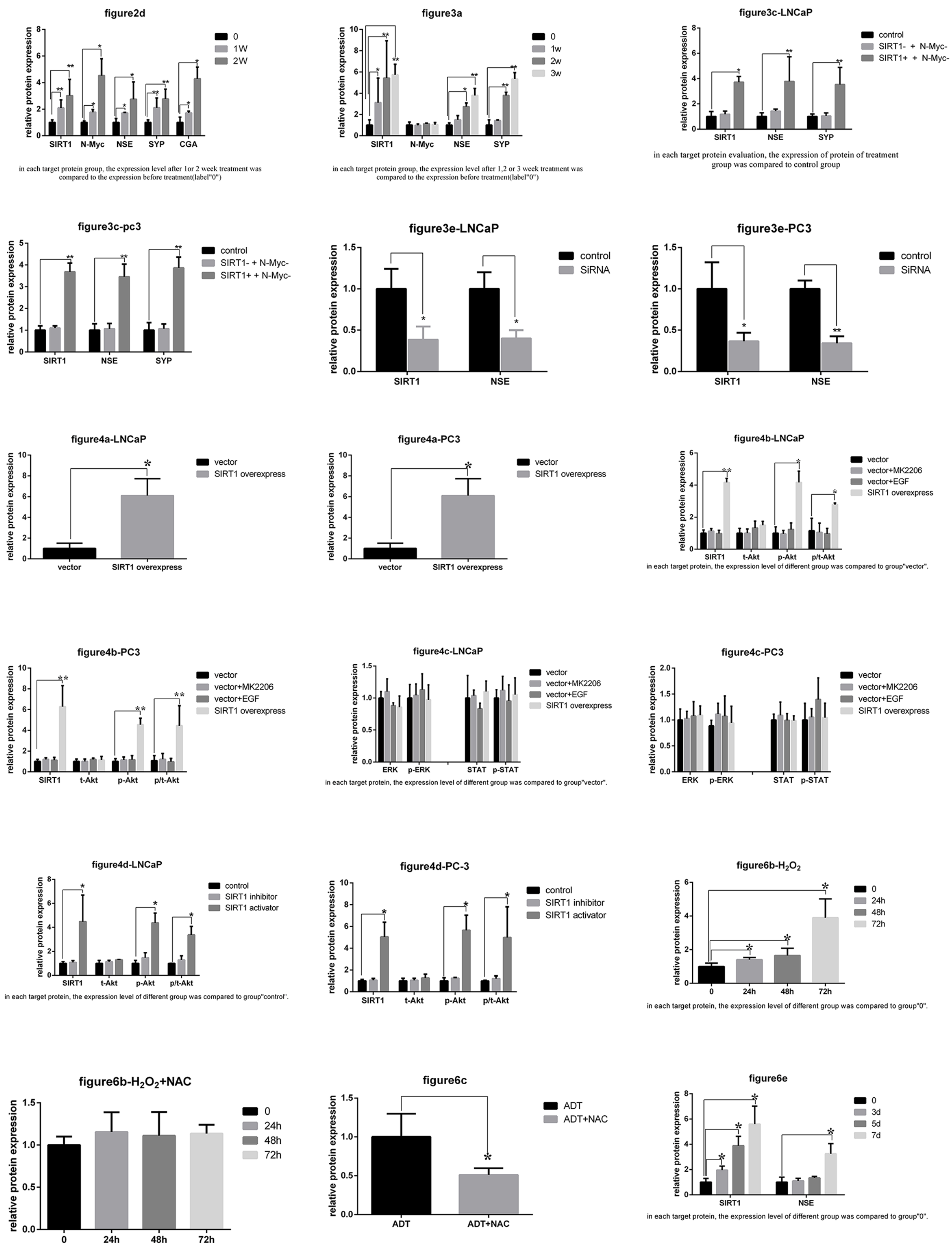

***, p<0.05; **, p<0.01**

Supplementary Figure 8: Quantitative results of the western blotting.

Supplementary Table 1: Expression of epigenetic factors in NED of LNCaP cells following ADT.

See Supplementary File 1
