# Supplementary material for: SIRT1 contributes to neuroendocrine differentiation of prostate cancer
Source: Oncotarget. 2017 Dec 11;9(2):2002–16. doi: 10.18632/oncotarget.23111 (PMC5788616; doi:10.18632/oncotarget.23111)
Supplement: Supplementary file 2 [file oncotarget-09-2002-s002.doc]

**Supplementary Table 1:** Expression of epigenetic factors in NED of LNCaP cells following ADT

| ID | Gene.symbol | FC | adj.P.Val | Gene.title |
| --- | --- | --- | --- | --- |
| 8124527 | HIST1H1B | 0.076788 | 1.52E-08 | histone cluster 1, H1b |
| 8030753 | KLK3 | 0.116759 | 1.52E-08 | kallikrein related peptidase 3 |
| 8014974 | TOP2A | 0.06389 | 3.25E-08 | topoisomerase (DNA) II alpha |
| 7983969 | CCNB2 | 0.067543 | 4.40E-08 | cyclin B2 |
| 8007071 | CDC6 | 0.089605 | 4.83E-08 | cell division cycle 6 |
| 8124531 | HIST1H3I | 0.063854 | 5.73E-08 | histone cluster 1, H3i |
| 8124388 | HIST1H3B | 0.077247 | 6.45E-08 | histone cluster 1, H3b |
| 8063043 | UBE2C | 0.236321 | 8.52E-08 | ubiquitin conjugating enzyme E2 C |
| 8106098 | MAP1B | 4.356787 | 9.22E-08 | microtubule associated protein 1B |
| 8117594 | HIST1H2BM | 0.076968 | 9.45E-08 | histone cluster 1, H2bm |
| 8034772 | ASF1B | 0.239979 | 1.36E-07 | anti-silencing function 1B histone chaperone |
| 8102643 | CCNA2 | 0.141494 | 1.51E-07 | cyclin A2 |
| 8171297 | MID1 | 3.041466 | 2.88E-07 | midline 1 |
| 8071212 | CDC45 | 0.227162 | 3.41E-07 | cell division cycle 45 |
| 7981525 | CDCA4 | 0.449833 | 8.02E-07 | cell division cycle associated 4 |
| 8143663 | EZH2 | 0.343442 | 8.38E-07 | enhancer of zeste 2 polycomb repressive complex 2 subunit |
| 8114425 | CDC25C | 0.224569 | 8.89E-07 | cell division cycle 25C |
| 8028084 | APLP1 | 2.132552 | 9.87E-07 | amyloid beta precursor like protein 1 |
| 7962579 | AMIGO2 | 3.08425 | 1.07E-06 | adhesion molecule with Ig like domain 2 |
| 8089527 | ATG3 | 2.963889 | 1.21E-06 | autophagy related 3 |
| 8142194 | LAMB1 | 2.185267 | 1.41E-06 | laminin subunit beta 1 |
| 8058765 | FN1 | 2.141374 | 1.49E-06 | fibronectin 1 |
| 7901192 | RAD54L | 0.376942 | 2.10E-06 | RAD54-like (S. cerevisiae) |
| 8056408 | GALNT3 | 2.297627 | 2.59E-06 | polypeptide N-acetylgalactosaminyltransferase 3 |
| 7986068 | BLM | 0.348246 | 4.78E-06 | Bloom syndrome RecQ like helicase |
| 8179184 | DDR1 | 1.692833 | 4.91E-06 | discoidin domain receptor tyrosine kinase 1 |
| 8133983 | ADAM22 | 1.672889 | 5.95E-06 | ADAM metallopeptidase domain 22 |
| 8076393 | CENPM | 0.455809 | 6.24E-06 | centromere protein M |
| 8083709 | SMC4 | 0.476161 | 6.24E-06 | structural maintenance of chromosomes 4 |
| 8082350 | MCM2 | 0.34996 | 7.67E-06 | minichromosome maintenance complex component 2 |
| 7927814 | SIRT1 | 1.63061 | 8.52E-06 | sirtuin 1 |
| 8103728 | HMGB2 | 0.280642 | 9.02E-06 | high mobility group box 2 |
| 8008237 | ITGA3 | 1.745829 | 9.35E-06 | integrin subunit alpha 3 |
| 7953626 | CLSTN3 | 1.52018 | 1.28E-05 | calsyntenin 3 |
| 8177222 | CD24 | 2.401458 | 1.46E-05 | CD24 molecule |
| 8024900 | UHRF1 | 0.461416 | 2.76E-05 | ubiquitin like with PHD and ring finger domains 1 |
| 7997839 | CDT1 | 0.61361 | 3.21E-05 | chromatin licensing and DNA replication factor 1 |
| 8108729 | PCDHB11 | 1.689077 | 4.79E-05 | protocadherin beta 11 |
| 8105842 | CENPH | 0.459665 | 5.16E-05 | centromere protein H |
| 8135601 | MET | 1.66354 | 9.28E-05 | MET proto-oncogene, receptor tyrosine kinase |
| 8081657 | CD200 | 1.973076 | 1.06E-04 | CD200 molecule |
| 8100734 | UGT2B17 | 1.679232 | 1.45E-04 | UDP glucuronosyltransferase family 2 member B17 |
| 8065710 | E2F1 | 0.620304 | 3.85E-04 | E2F transcription factor 1 |
| 7917649 | TGFBR3 | 1.443905 | 6.67E-04 | transforming growth factor beta receptor 3 |
| 8034578 | KLF1 | 0.795379 | 1.99E-03 | Kruppel like factor 1 |
| 7932966 | ITGB1 | 1.226719 | 2.20E-03 | integrin subunit beta 1 |
| 8004571 | EFNB3 | 0.824308 | 2.39E-03 | ephrin B3 |
| 8108708 | PCDHB7 | 1.561525 | 2.63E-03 | protocadherin beta 7 |
| 8007637 | FZD2 | 0.826892 | 3.29E-03 | frizzled class receptor 2 |
| 8145334 | ADAM7 | 0.803715 | 4.36E-03 | ADAM metallopeptidase domain 7 |
| 8090343 | KLF15 | 0.842187 | 5.10E-03 | Kruppel like factor 15 |
| 7949796 | GPR152 | 0.814354 | 6.39E-03 | G protein-coupled receptor 152 |
| 8055702 | NMI | 1.184936 | 7.41E-03 | N-myc and STAT interactor |
| 8030105 | FGF21 | 0.864935 | 8.24E-03 | fibroblast growth factor 21 |
| 8128284 | EPHA7 | 1.178349 | 8.55E-03 | EPH receptor A7 |
